# Hepatocyte-specific Smad4 deficiency inhibits hepatocarcinogenesis by promoting CXCL10/CXCR3-dependent CD8^+^- T cell-mediated anti-tumor immunity

**DOI:** 10.7150/thno.97276

**Published:** 2024-09-09

**Authors:** Xin Xin, Zhao Li, Xuanxuan Yan, Ting Liu, Zuyin Li, Zhuomiaoyu Chen, Xinlong Yan, Fanxin Zeng, Lingling Hou, Jinhua Zhang

**Affiliations:** 1 The College of Life Science and Bioengineering, Beijing Jiaotong University, Beijing, China.; 2 Department of Hepatobiliary Surgery, Peking University People's Hospital, Beijing, China.; 3 School of Life Science and Technology, Jinan University, Guangzhou, Guangdong province, China.; 4 Faculty of Environmental and Life Sciences, Beijing University of Technology, Beijing, China.; 5 Department of Clinical Research Center, Dazhou Central Hospital, Dazhou, Sichuan province, China.

**Keywords:** hepatocyte, Smad4, hepatocellular carcinoma, CXCL10, aerobic glycolysis

## Abstract

**Rationale:** Sma mothers against decapentaplegic homologue 4 (Smad4) is a key mediator of the transforming growth factor β (TGF-β) pathway and plays complex and contradictory roles in hepatocellular carcinoma (HCC). However, the specific role of Smad4 in hepatocytes in regulating hepatocarcinogenesis remains poorly elucidated.

**Methods:** A diethylnitrosamine/carbon tetrachloride-induced HCC model was established in mice with hepatocyte-specific Smad4 deletion (Alb^Smad4-/-^) and liver tumorigenesis was monitored. Immune cell infiltration was examined by immunofluorescence and fluorescence activated cell sorting (FACS). Cytokine secretion, glycolysis, signal pathway, and single-cell RNA sequencing were analysed for mechanism.

**Results:** Alb^Smad4-/-^ mice exhibited significantly fewer and smaller liver tumor nodules, less fibrosis, reduced myeloid-derived suppressor cell infiltration and increased CD8^+^ T cell infiltration. Smad4 deletion in hepatocytes enhanced C-X-C motif ligand 10 (CXCL10) secretion, promoting tumor necrosis factor-α (TNF-α) production in CD8^+^ T cells. The loss of Smad4 activated the CXCL10/mammalian target of rapamycin (mTOR)/lactate dehydrogenase A (LDHA) pathway, which increased glycolytic activity in CD8^+^ T cells. HCC patients with high Smad4 expression exhibited decreased CD8^+^ T cell infiltration and altered glycolysis.

**Conclusion:** Our results demonstrate that Smad4 in hepatocytes promotes hepatocarcinogenesis and is a potential and candidate target for the prevention and therapy of HCC.

## Introduction

Hepatocellular carcinoma (HCC) is the most prevalent primary liver cancer, accounting for approximately 90% of all cases, and is a leading cause of cancer-related deaths worldwide 1. Emerging evidence suggests that the aetiology of HCC is multifactorial. HCC most commonly occurs in people with chronic liver diseases, such as inflammation, fibrosis and cirrhosis caused by hepatitis B virus (HBV) or hepatitis C virus (HCV) infection, alcohol consumption, and metabolic syndrome 2, 3. The approval of new drugs, and the establishment of therapies based on immune checkpoint blockade, provide multiple treatment options for patients 4. Unfortunately, HCC remains a lethal malignancy with a five-year survival rate of only 21% 5. Therefore, it is important to better understand the signaling mechanisms in the HCC tumor microenvironment (TME) and to identify new targets for clinical anti-tumor therapy.

The HCC TME is a complex niche composed of tumor cells, infiltrating immune cells, cytokines, and chemokines, which collectively contribute to the immunosuppressive effects that in turn prompt HCC proliferation, invasion, and metastasis 6, 7. Within this environment, the interaction between tumor and immune cells, particularly CD8^+^ T cells, is critical for determining tumor progression and the response to therapy 8. Tumor cells produce many chemokines that recruit immune cells into the TME via specific chemokine receptors 6, 9. Metabolic reprogramming within the TME, including alterations in aerobic glycolysis, has been shown to be a key factor in regulating immune cell function and tumor progression 10.

The TGF-β signaling pathway plays important roles in cell proliferation, apoptosis, differentiation, migration, and anti-tumor immunity, and naturally plays a pivotal regulatory role in HCC progression 11-13. Smad4, the central mediator of TGF-β signaling, is also involved in key development processes of liver inflammation 14, fibrosis 15, fatty liver 16, and liver cancer 17. Although Smad4 is ubiquitously expressed across various cell types, its functional role is distinctly specific to each cell type. Smad4 in hepatocytes promotes inflammation and collagen deposition during the progression of non-alcoholic steatohepatitis (NASH) 14. We recently found that Smad4 deletion in hepatocytes alleviates liver fibrosis via the p38/p65 pathway 15. Smad4 deficiency in stellate cells has also been found to significantly reduce the expression level of fibrotic genes 18. Additionally, Smad4 upregulates the expression of genes that encode T-cell receptor (TCR) complex components and cytotoxic effector molecules in CD8^+^ T cells 19, 20. Smad4 deletion in natural killer (NK) cells leads to the impairment of NK cell maturation and homeostasis 21. In recent studies Smad4 expression was found to be upregulated in human HCC tumors and was correlated with poor postoperative prognosis in patients with HCC 22, 23. Conversely, a previous study has reported the presence of a lower protein level of Smad4 in HCC tissue compared with adjacent liver tissue in an Asian HCC cohort 24. To date, the role of Smad4 in hepatocytes during HCC development remains unclear.

In this study, we explored the role of Smad4 in hepatocytes during fibrosis-related hepatocarcinogenesis using hepatocyte-specific Smad4 knockout (Alb^Smad4-/-^) mice. The study demonstrated that hepatocyte-specific Smad4 deletion reduced tumor incidence after diethylnitrosamine (DEN) and carbon tetrachloride (CCl_4_) treatment. Moreover, Smad4 deletion in hepatocytes increased the secretion of C-X-C motif ligand 10 (CXCL10), which promoting tumor necrosis factor-α (TNF-α) production and glycolysis in CD8^+^ T cells.

## Methods

Some detailed information was provided in supplementary data. The details of RT-qPCR primers are described in supplementary material, Table S1.

### Tissue microarray immunohistochemistry staining

Tissue microarrays (TMAs) consist of 20 HCC specimens, 20 intrahepatic cholangiocarcinoma (ICC) specimens, 5 metastatic cancer specimens, 14 cirrhosis specimens, 11 hepatitis specimens, and 5 healthy liver control specimens (Taibsbio Technology, Xi'an, China). Smad4 expression was determined by immunohistochemistry (IHC) using a rabbit anti-Smad4 antibody (Affinity Biosciences, Cincinnati, OH, USA). The evaluation of Smad4 staining was carried out according to a method described in a previous study 25. The intensity of Smad4 expression was scored as follows: 0, negative; 1, weak; 2, moderate; 3, strong. The extent of staining was scored as follows: 1, 0 to <25%; 2, 25 to <50%; 3, 50 to <75%; or 4, 75 to <100%. Five randomly selected fields were observed under a light microscope. The final score was determined by multiplying the intensity scores by the extent of staining. Sums from 0 to 5 were defined as negative for Smad4; sums from >5 to 35 were defined as low expression of Smad4; and sums from >35 to 60 were defined as high expression of Smad4.

### Mice

Albumin-Cre (Alb-Cre) and Smad4 flox/flox (Smad4^fl/fl^) mice on a C57BL/6 background were purchased from Jackson Laboratory (Bar Harbor, ME, USA) 26. Mice with a conditional knockout of Smad4 in albumin-expressing hepatocytes (Alb^Smad4-/-^) were generated by crossing Smad4 flox/flox and Alb-cre mice. Cre-negative littermates were used as control mice. All mice were maintained in specific pathogen-free and humidity-and temperature-controlled microisolator cages with a 12-h light/dark cycle at the Institute of Biophysics, Chinese Academy of Sciences. All animal studies were performed after being approved by the Institutional Laboratory Animal Care and Use Committee of Beijing Jiaotong University.

### DEN/CCl_4_-induced HCC model

The mice were first treated with an intraperitoneal (i.p.) injection of 50 μg/g DEN (Sigma-Aldrich, St. Louis, MO, USA) at the age of 15 days. At the age of 8 weeks, mice were then treated with 0.5 μl/g body weight of CCl_4_, diluted (1:9) in corn oil by i.p. injection twice weekly for 6 weeks. Tumor development was monitored at 30 weeks as described previously 25.

### Histochemistry and immunostaining

Preparation of paraffin and cryostat tissue sections was performed as described previously 27. The sliced liver paraffin sections were then stained with hematoxylin and eosin (H&E) and sirius red. For immunohistochemistry, paraffin sections were incubated with primary antibodies (rabbit anti-Smad4, Affinity Biosciences, Cincinnati, OH, USA) followed by incubation with horseradish peroxidase (HRP)-conjugated secondary antibodies. For immunofluorescence detection, paraffin sections were incubated with anti-PCNA primary antibodies (Santa Cruz Biotechnology, Shanghai, China), while cryostat sections were incubated with anti-F4/80, anti-CD11b, and anti-Gr-1 primary antibodies (BD Pharmingen, San Diego, CA, USA), respectively, and followed by incubation with Alexa Fluor 488- or 594-conjugated secondary antibodies (Invitrogen, Carlsbad, CA, USA). Cell nuclei were stained with DAPI. Sections were evaluated under a microscope (DP71, Olympus, Tokyo, Japan) for bright-field and fluorescence microscopy.

### Isolation and activation of naïve CD8^+^ T lymphocytes

Naïve CD8^+^ T lymphocytes were isolated from mouse spleens by negative selection using the Naïve CD8^+^ T Cell Isolation Kit (BioLegend, USA). Following isolation, naïve CD8^+^ T lymphocytes were activated with plate-bound 2 µg/mL anti-CD3 (BioLegend, USA) and 1 µg/mL anti-CD28 (BioLegend, USA) and cultured in RPMI-1640 medium supplemented with 10% heat-inactivated FBS (Gibco, Grand Island, NY, USA), 10 mM HEPES (BI, Israel), 0.05 mM β-mercaptoethanol (BI, Israel), and 1% penicillin-streptomycin. For intracellular cytokine staining, CD8^+^ T cells were stimulated with PMA/ionomycin mixture (Multisciences Biotech, Co., Ltd., Hangzhou, China) and BFA/monensin mixture (Multisciences Biotech, Co., Ltd., Hangzhou, China) for 6 h.

### Seahorse assays

Extracellular acidification rate (ECAR) and oxygen consumption rate (OCR) were measured with a XFe96 Extracellular Flux Analyzer (Agilent Technologies) following protocols recommended by the manufacturer. CD8^+^ T cells were isolated from the spleens and activated with 2 μg/mL anti-CD3 and 1 μg/mL anti-CD28 for 48 h. CXCL10 treated CD8^+^ T cells with or without AMG487 were seeded on XFe96 microplates that had been pre-coated with Cell-Tak adhesive (BD Biosciences). The plates were quickly centrifuged to immobilize cells. Cells were rested in a non-buffered assay medium for 30 min before starting the assay. Glycolysis or oxidative phosphorylation (OXPHOS) associated parameters were measured by Seahorse XFe Glycolysis Stress test kit (Agilent Technologies). In a glycolysis assay, three compounds are injected separately: 10 mM glucose, 1 μM oligomycin, and 50 mM 2-deoxyglucose (2-DG). In an OXPHOS assay, three compounds are injected separately: 2 μM oligomycin, 2 μM carbonyl cyanide p-(trifluoromethoxy) phenylhydrazone (FCCP), and a combination of 1 μM antimycin A and 1 μM rotenone.

### Single-cell RNA sequencing analysis

Eighty samples of human HCC were analyzed by single-cell RNA sequencing (scRNA-seq), The scRNA-seq approach utilized in this study was previously described in detail 28. Patients were then ranked based on the mean expression level of Smad4 in their tumor cells and divided into two groups: Smad4-high and Smad4-low.

### Statistical analysis

All data were showed as the mean ± SEM and analyzed using GraphPad Prism V8.0.2 software. Differences between two groups were compared using two-tailed unpaired Student's t-test analysis. Two-way ANOVA was used for multiple comparisons. P < 0.05 was considered statistically significant.

## Results

### Smad4 is highly expressed in fibrosis-related HCC

**Figure 1:**
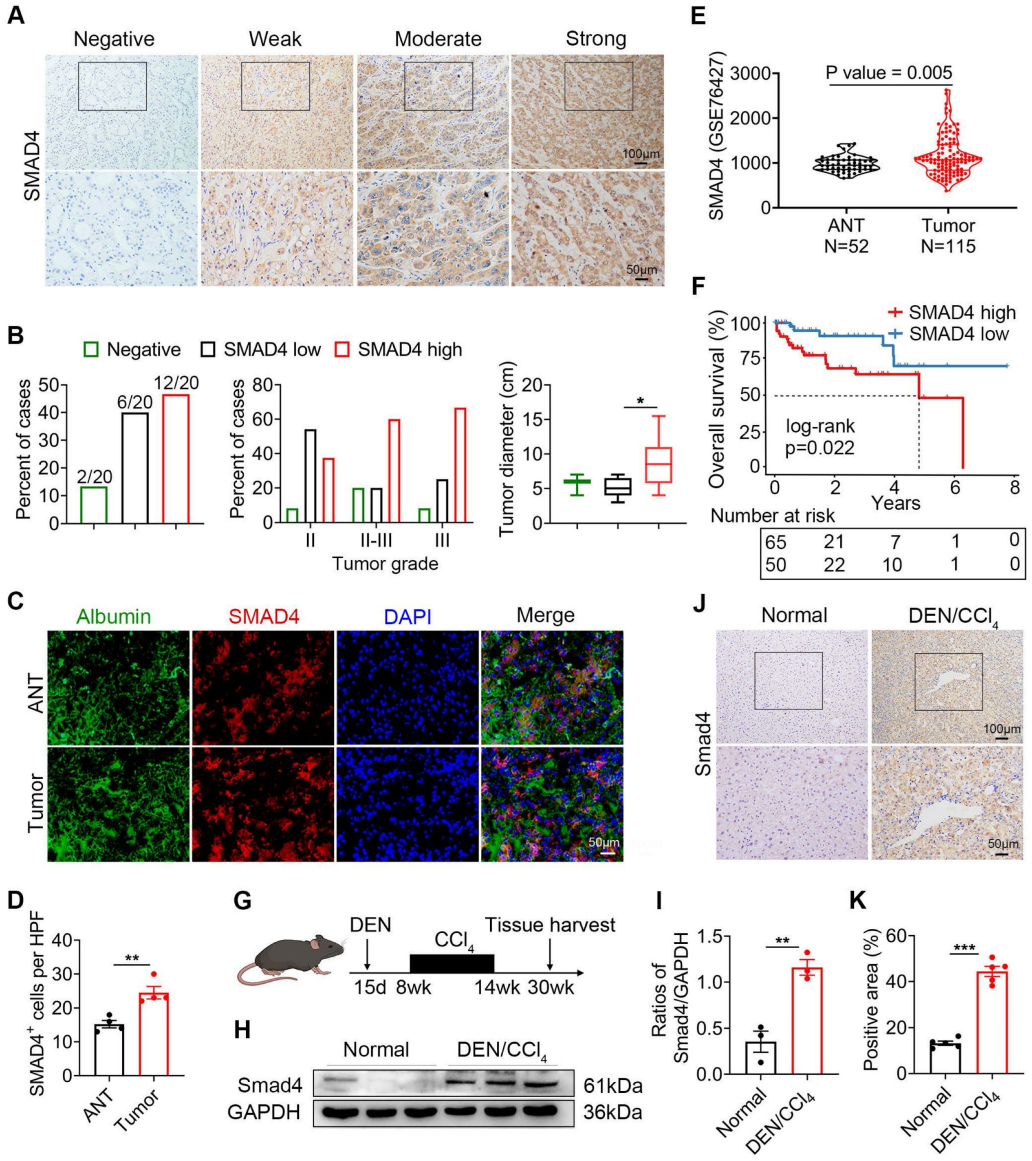
** Smad4 expression is upregulated in human HCC and DEN/CCl_4_ -induced mouse HCC.** (A-E) Immunohistochemical staining for Smad4 in HCC patients. (A) Representative IHC images of Smad4 in a tissue microarray from 20 HCC patients. Scale bar: 50 μm. (B) Percentage of the cases expressing Smad4 in carcinoma tissues (left), percentage of tissues with negative, low, and high Smad4 expression with different tumor grades (middle) and average tumor diameter of different Smad4 protein levels in HCC patients (right). *P < 0.05. (C-D) Representative double staining of albumin (green) and Smad4 (red) in adjacent non-tumor tissues (ANT) and tumor tissues from human HCC. Scale bar: 50 μm. (E) GSE 76427 dataset was used to analyze the difference in Smad4 expression between ANT and tumor tissues. (F) Kaplan-Meier survival analysis of the Smad4 low and high expression in the GSE 76427 datasets. (G) Schematic representation of the DEN/CCl_4_-induced liver fibrosis-related HCC model. (H-I) Western blot analysis of Smad4 protein levels in HCC tissues. Smad4 expression was normalized to the Normal GAPDH. **P < 0.01. (J-K) Representative immunohistochemical staining for Samd4 in mice normal liver and HCC tissues. Scale bar: 50 μm. ***P < 0.001.

We previously demonstrated that Smad4 deficiency in hepatocytes alleviated CCl_4_-treated liver fibrosis 15. In human tissue microarray (TMA) analysis, Smad4 expression significantly increased in cirrhosis and HCC specimens compared with healthy liver specimens (Figure S1A-B). Interestingly, HCC liver exhibited a significantly higher percentage of nuclear positive Smad4 compared with healthy, hepatitis and cirrhotic livers (Figure S1C). To further detect Smad4 expression in fibrosis-related HCC, TMAs from 20 patients with HCC were used for immunohistochemical staining (Figure 1A), and approximately 10% of the cases were negative for Smad4, and 30% and 60% had low or high Smad4 expression respectively (Figure 1B). There was a positive correlation between the increased Smad4 expression and tumor grades, indicating that the Smad4 expression was higher in patients with advanced HCC (Figure 1B). Furthermore, patients with high Smad4 expression had a significantly larger tumor diameter (Figure 1B). Subsequent double immunofluorescence staining revealed that Smad4 was highly expressed in albumin^+^ cells in HCC tissues (Figure 1C-D). In addition, HCC patients with high Smad4 expression had shorter survival time by analysis of the GEO database (Figure 1E-F).

To further explore the role of Smad4 in HCC, we established a mouse fibrosis related liver cancer model using DEN/CCl_4_ treatment (Figure 1G). Immunohistochemical and western blot analysis demonstrated that Smad4 expression in tumor tissues was significantly higher than in normal tissues (Figure 1H-K). Consistently, double immunofluorescence staining indicated that Smad4 was highly expressed in albumin^+^ hepatocytes in mouse HCC tumor tissues (Figure S1D). These results demonstrated that Smad4 expression was closely correlated with fibrosis-related HCC.

### Hepatocyte-specific Smad4 deletion alleviates DEN/CCl_4_-induced fibrosis-related hepatocarcinogenesis

**Figure 2:**
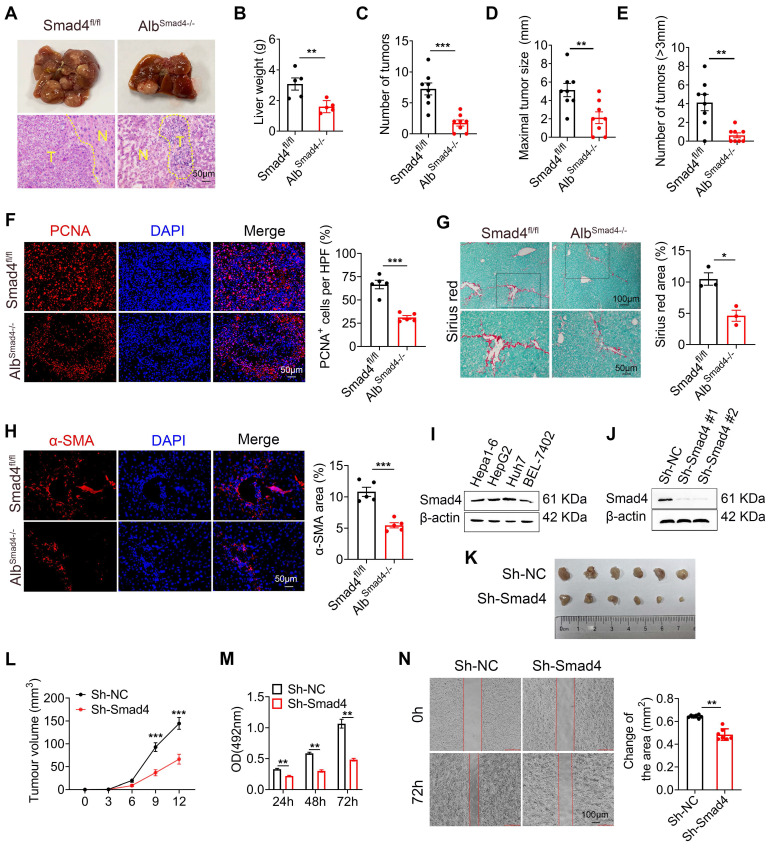
** Smad4 deletion in hepatocytes alleviates DEN/CCl_4_-induced hepatocarcinogenesis and tumor cell proliferation.** (A-E) Groups of Smad4^fl/fl^ and Alb^Samd4-/-^ mice (n = 8 per group) were used for the DEN/CCl_4_-induced HCC model. (A) Gross morphology (top) and H&E staining (bottom) of the livers of the Smad4^fl/fl^ and Alb^Samd4-/-^ mice. N, normal liver tissue. T, liver tumor area. Scale bar: 50 μm. (B) Liver weight per mouse, (C) number of tumors per mouse, (D) size of the tumors, and (E) number of >3mm tumors per mouse in Smad4^fl/fl^ and Alb^Samd4-/-^ mice are shown. **P < 0.01 and ***P < 0.001. (F) Representative staining of PCNA in HCC tissues (Scale bars: 50 μm) and statistical analysis. ***P < 0.001. (G) Sirius red staining of liver tissues in Smad4^fl/fl^ and Alb^Samd4-/-^ mice (Scale bars: 100 μm, zoom in: 50 μm), quantification of stained areas and statistical analysis. *P < 0.05. (H) Immunofluorescence staining of α-SMA in HCC tissues (Scale bars: 50 μm) and statistical analysis. ***P < 0.001. (I) Western blot analysis of Smad4 protein levels in Hepa1-6, HepG2, Huh7 and BEL-7402 cell lines. (J) The characterization of Smad4 in sh-NC and sh-Smad4 Hepa1-6 cells by western blot. (K) *Ex vivo* images of resected tumors (Scale bars: 1 cm). (n = 6 per group). (L) growth curves of tumor volume formed by subcutaneous injection of Hepa1-6 cells (n = 6 per group). ***P < 0.001. (M) The proliferation ability of Hepa1-6 cells at 24 h, 48 h and 72 h. **P < 0.01. (N) Representative photographs of wound-healing assay and statistical analysis. Hepa1-6 cells were scratched using pipet tips for 72 h. The migration ability of Hepa1-6 cells was evaluated. **P < 0.01.

To investigate the function of Smad4 in hepatocytes in HCC, transgenic mice expressing Cre recombinase from the albumin promoter were crossed with Smad4^fl/fl^ mice to establish hepatocyte-specific Smad4 knockout mouse (Alb^Smad4-/-^). Smad4 deletion in hepatocytes from Alb^Smad4-/-^ mice was confirmed by double immunofluorescence staining of albumin and Smad4 (Figure S1E). Alb^Smad4-/-^ and Smad4^fl/fl^ mice were given a single intraperitoneal injection of DEN, followed by CCl_4_ treatment twice weekly for 6 weeks, and liver tumorigenesis was monitored for 30 weeks. The tumor morphology and H&E staining indicated the successful induction of HCC by DEN/CCl_4_ (Figure 2A). All Smad4^fl/fl^ mice developed liver tumors within 30 weeks. However, Alb^Smad4-/-^ mice showed obvious resistance to hepatocarcinogenesis (Figure 2A). Hepatocyte-specific Smad4 deletion significantly decreased the number and size of HCC tumors (Figure 2B-E). Cell proliferation was also significantly weakened in Smad4-deficient tumors by proliferating cell nuclear antigen (PCNA) staining (Figure 2F). Moreover, Sirius red staining and α-smooth muscle actin (α-SMA) immunofluorescence staining revealed an attenuated fibrosis level in Alb^Smad4-/-^ mice (Figure 2G-H). To further confirm the role of Smad4 in liver tumor development, we detected the Smad4 expression in different human and murine HCC cell lines and found that Smad4 was expressed in these cell lines (Figure 2I). Then Smad4 expression in Hepa1-6 cells was knocked down by a Smad4-targeting lentiviral vector and the level of Smad4 was assessed by western blot (Figure 2J). Negative control (sh-NC) and sh-Smad4 Hepa1-6 cells were transplanted into C57BL/6 mice and Hepa1-6 cells with Smad4 deficiency developed smaller tumors than the sh-NC group (Figure 2K-L). *In vitro*, 3-(4,5-dimethylthiazol-2-yl)-2,5-diphenyltetrazolium bromide (MTT), wound-healing assays and western blot analysis of PCNA also showed that Smad4 deletion remarkably inhibited Hepa1-6 proliferation and migration (Figure 2M-N, Figure S2A). These results demonstrated that Samd4 in hepatocytes promoted fibrosis-related HCC development.

### Samd4 deletion in hepatocytes reduces MDSC infiltration and enhances CD8^+^ T cell infiltration in HCC

**Figure 3:**
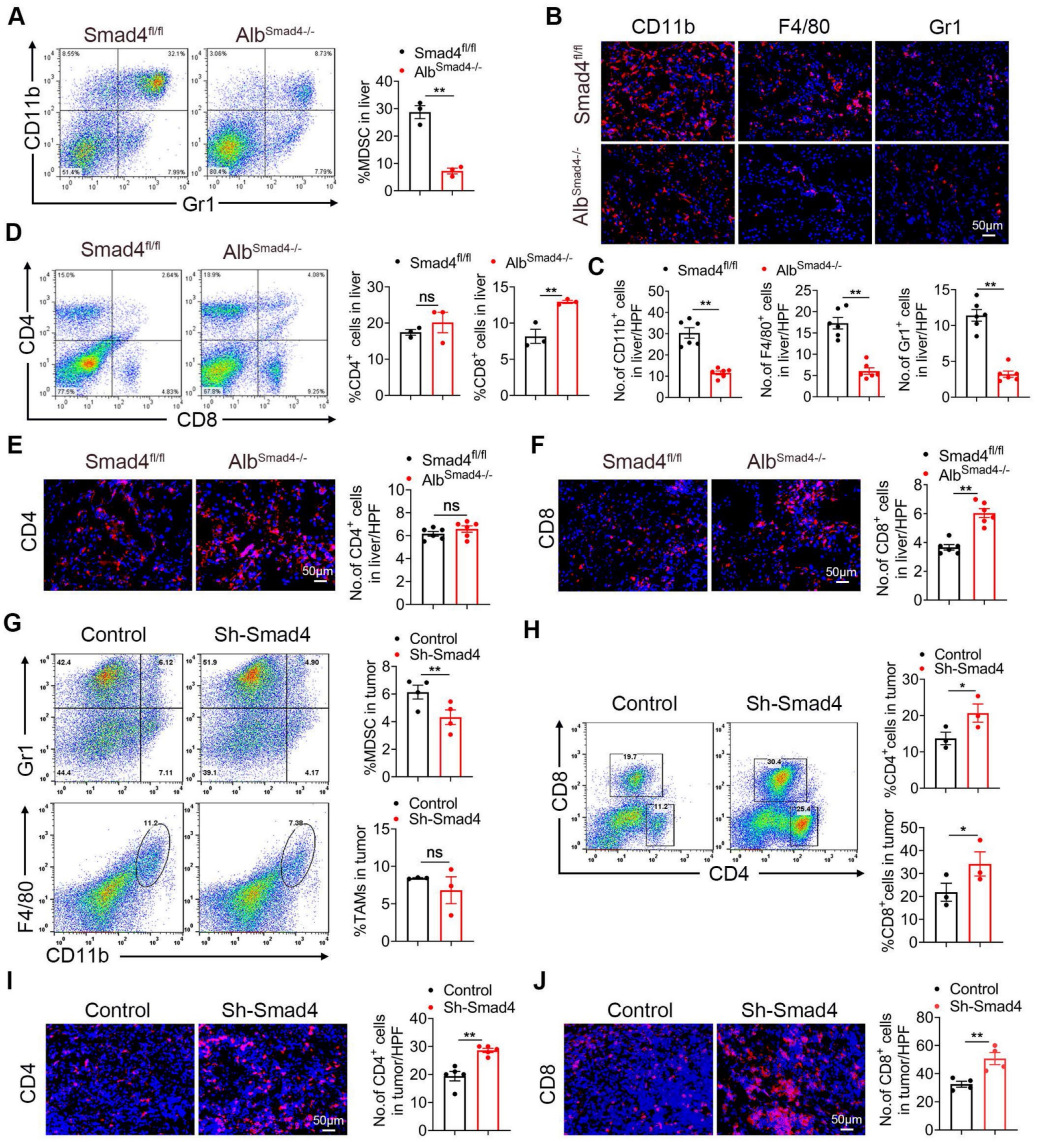
** Smad4 deletion in hepatocytes enhances the CD8^+^ T cell infiltration in HCC.** (A-F) Groups of Smad4^fl/fl^ and Alb^Samd4-/-^ mice were used for the CCl_4_-induced liver fibrosis model (n = 6 per group) and DEN/CCl_4_-induced HCC model (n = 8 per group). (A) Isolation of liver lymphocytes from CCl_4_-induced Smad4^fl/fl^ and Alb^Samd4-/-^ mice and flow cytometry analysis of the proportion of CD11b^+^ Gr-1^+^ MDSC in the livers. **P < 0.01. (B-C) Representative staining and statistical analysis of CD11b^+^, F4/80^+^, and Gr-1^+^ cells in DEN/CCl_4_-induced HCC tissues. Scale bars: 50 μm. **P < 0.01. (D) Representative image of CD4^+^ and CD8^+^ proportion in fibrotic liver tissues analyzed by FACS and statistical analysis, respectively. **P < 0.01. (E-F) Representative staining of CD4^+^ and CD8^+^ cells in HCC liver tissues and statistical analysis, respectively. (Scale bars: 50 μm). **P < 0.01. (G) Representative images of FACS and statistical analysis for MDSC and TAMs cells proportion in Hepa1-6 transplanted tumors, sh-Smad4 vs. sh-NC. **P < 0.01. (H) Representative images of FACS and statistical analysis for CD4^+^ and CD8^+^ T cells proportion in Hepa1-6 transplanted tumors, sh-Smad4 vs. sh-NC. *P < 0.05. (I-J) Immunofluorescence detection and statistical analysis of CD4^+^ and CD8^+^ cells in Hepa1-6 transplanted tumors, sh-Smad4 vs. sh-NC, respectively. **P < 0.01.

To examine whether Smad4 is involved in immune cell infiltration in the TME, we analyzed immunocyte profiles in CCl_4_-induced liver fibrosis and DEN/CCl_4_-induced HCC tissues. Fluorescence-activated cell sorting (FACS) analysis showed that the percentages of myeloid-derived suppressor cells (MDSC) decreased significantly in CCl_4_-treated Alb^Smad4-/-^ mice (Figure 3A). Consistently, CD11b^+^, Gr1^+^ and F4/80^+^ cell infiltration was prominently reduced in liver tissues of Alb^Smad4-/-^ mice compared to Smad4^fl/fl^ mice in the DEN/CCl_4_-induced HCC model (Figure 3B-C). In addition, the number of CD11b^+^ /Gr1^+^ cells also decreased in Alb^Smad4-/-^ mice detected by double immunofluorescence staining (Figure S2B). Moreover, an increased CD8^+^ T cell infiltration in Alb^Smad4-/-^ mice liver (Figure 3D-F) was detected by FACS and immunofluorescence, suggesting that Smad4 deletion changed the immune microenvironment. However, there were no significant differences in the number of CD4^+^ T cells between the fibrotic liver tissues of Alb^Smad4-/-^ and Smad4^fl/fl^.

To investigate the role of Smad4 in anti-tumor T-cell responses, Hepa1-6 cells treated with sh-Smad4 or sh-NC were subcutaneously into C57BL/6 mice, and the immune cells in transplanted tumors were detected by FACS. MDSC infiltration was significantly reduced in tumors with Smad4 knockdown. There were no significant differences in tumor-associated macrophages (TAMs) infiltration between the sh-NC and sh-Smad4 groups (Figure 3G). Meanwhile, FACS analysis also showed that the proportion of CD4^+^ and CD8^+^ T cells increased in the sh-Samd4 group compared with sh-NC group (Figure 3H). Consistently, immunofluorescence staining also revealed similar results (Figure 3I-J). Furthermore, sh-NC and sh-Smad4 Hepa1-6 cells were inoculated subcutaneously in nude mice and Smad4 deficiency in Hepa1-6 cells didn't inhibit tumor growth compared with the sh-NC group, indicating that the anti-tumor effects of Smad4 mainly depended on the host's T cells, but not tumor cells (Figure S2C-D). Taken together, Smad4 deficiency in hepatocytes promotes the infiltration of CD8^+^ T cell along with the decrease of MDSCs in transplanted tumors, DEN/CCl_4_-induced HCC tumors and CCl_4_-induced fibrotic livers, suggesting that Smad4 promotes immune suppression in the HCC TME.

### Hepatocyte-derived CXCL10 was critical for TNF-α production of CD8^+^ T cells

**Figure 4:**
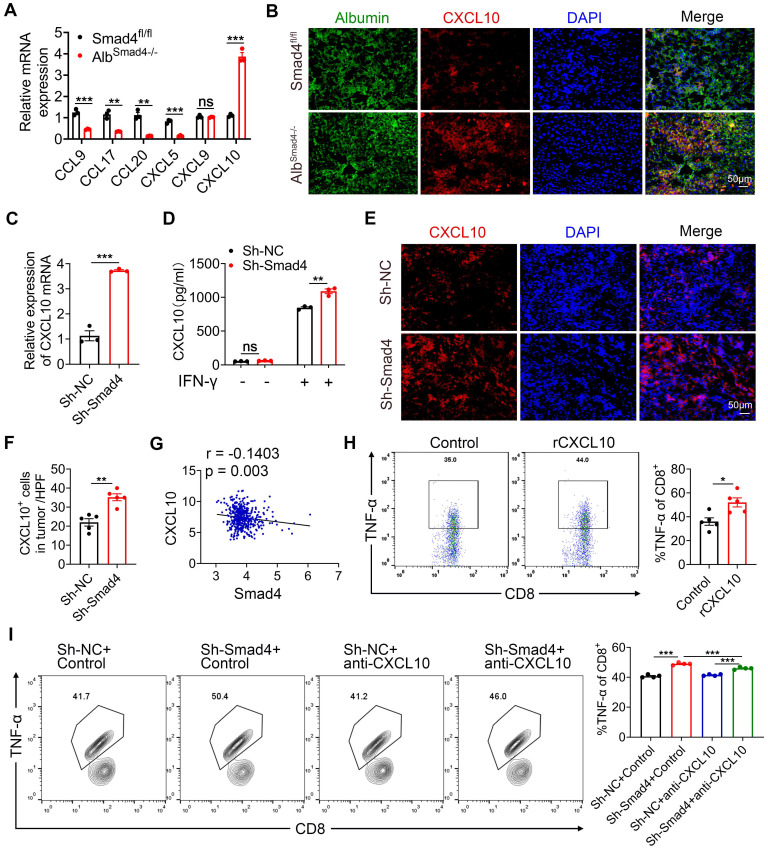
** Hepatocyte-derived CXCL10 was critical for TNF-α secretion in CD8^+^ T cells.** (A) The mRNA levels of CCL9, CCL17, CCL20, CXCL5, CXCL9 and CXCL10 in HCC tissues from Smad4^fl/fl^ and Alb^Samd4-/-^ mice were measured using RT-qPCR. **P < 0.01 and ***P < 0.001. (B) Groups of Smad4^fl/fl^ and Alb^Samd4-/-^ mice (n = 8 per group) were used as DEN/CCl_4_ HCC models. Representative double staining for albumin (green) and CXCL10 (red) in liver specimens. (Scale bar: 50 μm). (C) The mRNA levels of CXCL10 in Hepa1-6 cells with sh-Smad4 vs. sh-NC. ***P < 0.001. (D) Hepa1-6 cells were treated with IFN-γ (1 μg/ml) for 24 h. ELISA was performed to examine the levels of CXCL10. **P < 0.01. (E-F) Immunofluorescence staining of CXCL10 in Hepa1-6 tumor tissues (Scale bars: 50 μm) and statistical analysis. **P < 0.01. (G) Scatter plots show the negative correlation between Smad4 and CXCL10 mRNA expression in HCC GEO dataset (GSE 14520). Pearson's coefficient tests are performed to assess statistical significance. (H) Intracellular TNF-α levels of CD8^+^ T cells stimulated with PMA/Ionomycin and BFA/Monensin mixtures for 6 h in the presence of CXCL10 (100 ng/ml) and statistical analysis. *P < 0.05. (I) Hepa1-6 cells were co-cultured with pretreated CD8^+^ T cells at 1:3 in the absence or presence of an anti-CXCL10 neutralizing antibody (20 μg/ml) or control antibody in 24-well plates for 24 hours. The TNF-α levels in CD8^+^ T cells were identified by FACS. ***P < 0.001.

Hepatocytes in the TME secrete an array of chemokines to recruit immune cells, thereby promoting or suppressing tumor growth 29, 30. Chemokines chemokine C-C motif ligand (CCL) 9 (CCL9), CCL17, CCL20, CXCL5, CXCL9, and CXCL10 secreted by hepatocytes regulated tumor progression by acting on CD8^+^ T cells 31. To investigate the effects of Smad4 on chemokine secretion in hepatocytes, we detected the expression of CCL9, CCL17, CCL20, CXCL5, CXCL9 and CXCL10 in liver tissues from Smad4^fl/fl^ and Alb^Smad4-/-^ HCC mice by RT-qPCR. Results demonstrated that Smad4 knockout significantly increased the mRNA level of chemokine CXCL10, whereas CCL9, CCL17, CCL20, and CXCL5 levels were significantly reduced. There was no significant difference in CXCL9 expression (Figure 4A). Consistent with this, CXCL10 expression in hepatocytes was upregulated in DEN/CCl_4_-induced Alb^Smad4-/-^ mice by double immunofluorescence staining (Figure 4B). *In vitro*, the mRNA and protein levels of CXCL10 were further confirmed by RT-qPCR and ELISA. Smad4 knockdown in Hepa1-6 cells significantly facilitated the CXCL10 expression (Figure 4C-D). Consistent with the above results, the CXCL10 level was also increased significantly in sh-Smad4 Hepa1-6 transplanted tumors (Figure 4E-F). Additionally, we analyzed the correlation between Smad4 and CXCL10 expression in HCC using the GEO database (GSE 14520). As predicted, Smad4 expression negatively correlated with CXCL10 (Figure 4G).

CD8^+^ T cells are the main component of the anti-tumor immune response, eliminating target cells through exocytosis of effector cytokines such as granzyme B (Gzmb), interferon (IFN)-γ and TNF-α 32. To assess the effects of CXCL10 on anti-tumor immunity, we examined the effects of CXCL10 on the production of cytotoxic proteins and effector cytokines in mouse CD8^+^ T cells. CD8^+^ T cells were purified from naïve mouse spleens and stimulated with PMA/Ionomycin and BFA/Monensin mixtures in the presence of 100 ng/ml CXCL10 recombinant protein 33, and the production of TNF-α, IFN-γ, Gzmb, IL-2 was detected. FACS validated that the proportion of TNF-α in CD8^+^ T cells was significantly increased in the recombinant CXCL10 treatment group, but there was no difference in the levels of IFN-γ, Gzmb and IL-2 (Figure 4H, Figure S3A-C). However, these differences were not observed in CD4^+^ T cells (Figure S3D-F). Similarly, when CD8^+^ T cells were co-cultured with sh-Smad4 Hepa1-6 cells, the anti-CXCL10 neutralizing antibody significantly decreased TNF-α production in CD8^+^ T cells (Figure 4I). Thus, Smad4 in hepatocytes regulated CD8^+^ T cell TNF-α production through CXCL10.

### CXCL10 increases glycolysis in CD8^+^ T cells

**Figure 5:**
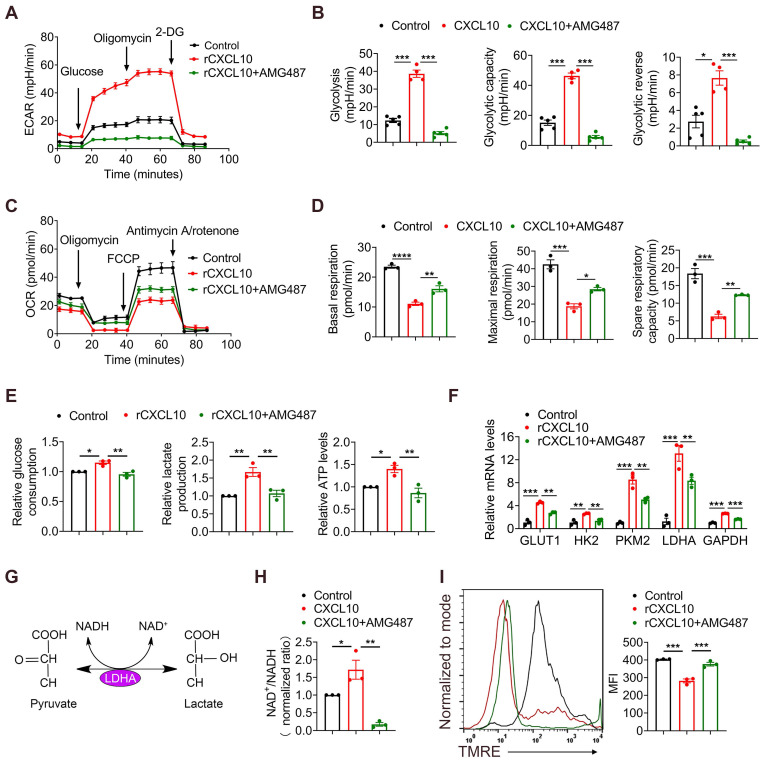
** CXCL10 promotes glycolysis and inhibits OXPHOS in CD8^+^ T cells.** CD8^+^ T cells were purified from naïve mouse spleens and cultured in anti-CD3/CD28-coated plates in the presence of CXCL10 (100 ng/ml) with or without AMG487 (5 μM) for 48 h. (A-B) Splenic CD8^+^ T cells were treated with anti-CD3 and anti-CD28 in the presence of CXCL10 (100 ng/ml) with or without AMG487 (5 μM) for 48 h, and a glycolytic stress test kit was used to measure the key parameters of glycolysis, and the extracellular acidification rate (ECAR) profile, glycolysis, glycolytic capacity, and glycolytic reserve were quantified. *P < 0.05 and ***P < 0.001. (C-D) Splenic CD8^+^ T cells were treated with anti-CD3 and anti-CD28 in the presence of CXCL10 (100 ng/ml) with or without AMG487 (5 μM) for 48 h, and a cell mito stress test kit was used to measure the key parameters, and the oxygen consumption rate (OCR) profile, basal respiration, maximal respiration, and spare respiratory capacity were quantified. *P < 0.05, **P < 0.01 and ***P < 0.001. (E) Relative glucose consumption, lactate production ratio and ATP levels of CD8^+^ T cells after a 48 h-long treatment with 100 ng/ml CXCL10, and with or without AMG487 (5 μM). *P < 0.05 and **P < 0.01. (F) RT-qPCR analysis of GLUT1, HK2, PKM2, LDHA and GAPDH expression in CD8^+^ T cells after a 48 h treatment with CXCL10 (100 ng/ml) and with or without AMG487 (5 μM). Data are presented as the means ± SEM from three independent experiments. Data in RT-qPCR analysis is normalized to control CD8^+^ T cells. **P < 0.01 and ***P < 0.001. (G) Schematic of LDH reaction. (H) Relative NAD^+^/NADH ratio of CD8^+^ T cells after a 48 h treatment with 100 ng/ml CXCL10, and with or without AMG487 (5 μM). *P < 0.05 and **P < 0.01. (I) Left, mitochondrial membrane potential as assessed by TMRE fluorescence in CD8^+^ T cells treated with 100 ng/ml CXCL10, 5 μM AMG487, or control for 48 h. Right, quantification of mean fluorescence intensity for TMRE. ***P < 0.001.

Accumulating evidence has shown that glycolytic metabolism plays a crucial role in the effector phase of CD8^+^ T cells 34. Therefore, we investigated whether CXCL10 affects the anti-tumor effects of CD8^+^ T by regulating their glycolytic metabolism. CD8^+^ T cells were purified from the spleen and stimulated with exogenous CXCL10 with or without AMG487 (CXCL10 receptor inhibitor (CXCR3)). To assess their metabolic functions, extracellular acidification rate (ECAR) and oxygen consumption rate (OCR) were measured for glycolysis and oxidative phosphorylation (OXPHOS), respectively, by a seahorse assay. Results demonstrated that CD8^+^ T cells treated by CXCL10 exhibited higher ECAR than untreated CD8^+^ T cells, evidenced by increased glycolytic capacity and reserve in CXCL10-stimulated CD8^+^ T cells. Blocking the effects of CXCL10 through receptor inhibitors significantly suppressed this phenomenon (Figure 5A-B). In contrast, the basal OCR, maximal respiration, and spare respiratory capacity of CD8^+^ T cells treated by CXCL10 were lower than those of the control group (Figure 5C-D). Furthermore, recombinant CXCL10 promoted glucose consumption, lactate and ATP production in CD8^+^ T cells (Figure 5E).

Glycolysis can enhance the conversion of pyruvate to lactate in glucose-rich conditions, resulting in increased glycolytic enzymes expression and NAD^+^ regeneration 35. We next examined the transcriptional profile of CD8^+^ T cells after CXCL10 stimulation using RT-qPCR. Consistently, the expression of key glycolysis genes, such as glucose transporter type 1 (GLUT1), hexokinase 2 (HK2), pyruvate kinase muscle isoenzyme 2 (PKM2), lactate dehydrogenase A (LDHA) and glyceraldehyde-3-phosphate dehydrogenase (GAPDH), was increased in CXCL10-treated CD8^+^ T cells compared to control cells, and AMG487 significantly suppressed this phenomenon (Figure 5F). Interestingly, the expression of LDHA increased the most. Studies have shown that LDHA can catalyze the interconversion of pyruvate to lactate and is accompanied by NAD^+^ regeneration 36, 37 (Figure 5G). We found that the NAD^+^/NADH ratio increased after treating CD8^+^ T cells with recombinant CXCL10, suggesting that LDHA activity was increased (Figure 5H). LDHA activation leads to less NADH accumulation in the cytoplasm, and NADH can enter the mitochondria and alter mitochondrial membrane potential (MMP) 38. Therefore, mitochondrial function was further detected by measuring MMP. A significant decrease of MMP was found in the CXCL10-stimulated group compared to control CD8^+^ T cells by measuring the fluorescence intensity of tetramethylrhodamine ethyl ester (TMRE) using FACS (Figure 5I). Thus, our results indicated that CXCL10 promoted LDHA activity in CD8^+^ T cells, resulting in altered glycolytic flux and NAD(H) balance.

### Mammalian target of rapamycin (mTOR) and LDHA inhibition reverses the effects of CXCL10 on CD8^+^ T cell metabolism and TNF-α production

**Figure 6:**
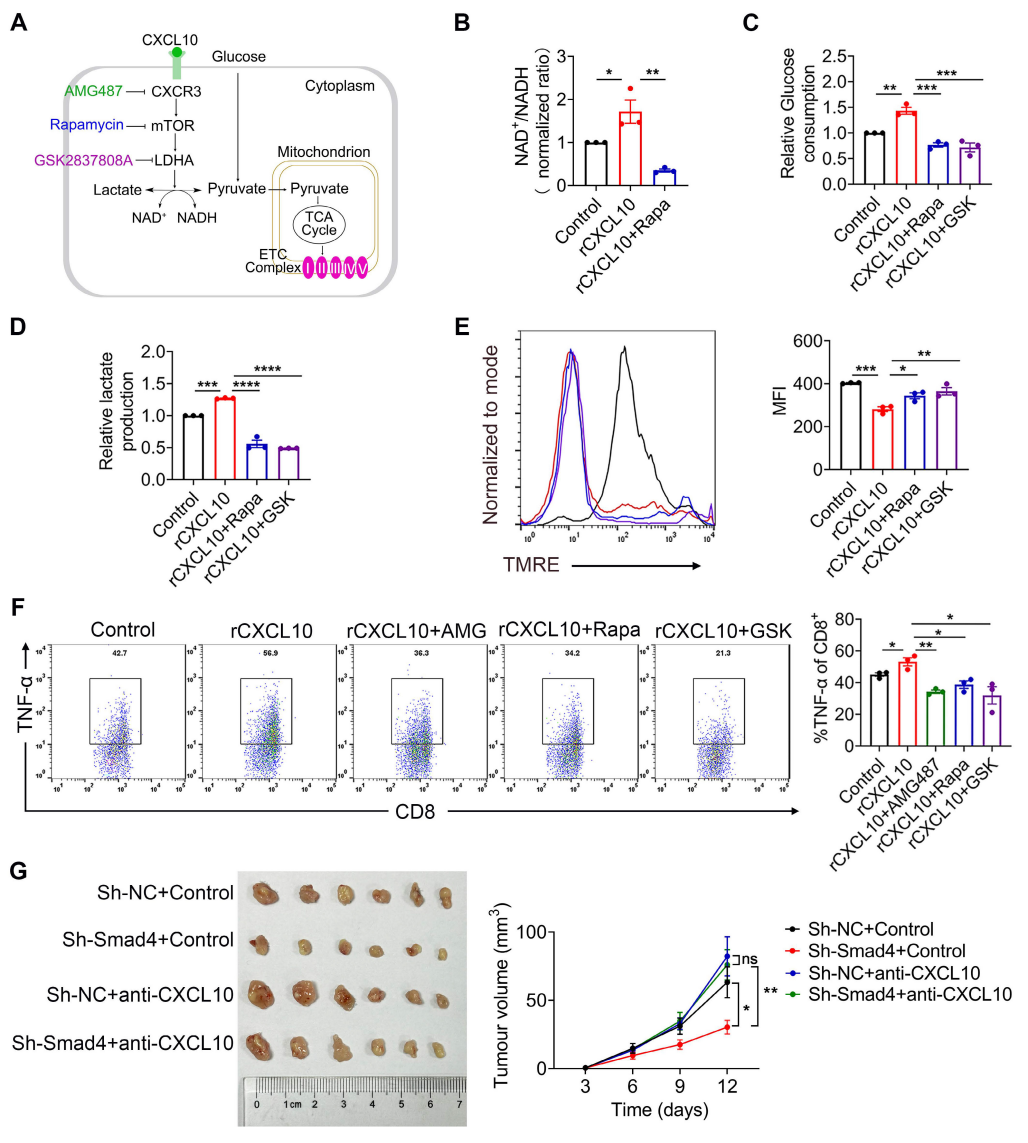
** mTOR and LDH inhibition recapitulates the effects of CXCL10 on CD8^+^ T cell metabolism and TNF-α production.** CD8^+^ T cells were purified from naïve mouse spleens and cultured in anti-CD3/CD28-coated plates in the presence of CXCL10 (100 ng/ml) with or without AMG487 (5 μM), Rapamycin (25 nM), and GSK2837808A (10 μM) for 48h. (A) Schematic of targets of CXCR3, mTOR, and LDHA. (B) NAD^+^/NADH ratio in CD8^+^ T cells treated with 100 ng/ml CXCL10, 25 nM Rapamycin, 10 μM GSK2837808A, or left untreated for 48 h. *P < 0.05 and **P < 0.01. (C) Relative glucose consumption (D) lactate production in CD8^+^ T cells treated with 100 ng/ml CXCL10, 25 nM Rapamycin, 10 μM GSK2837808A, or left untreated for 48 h. **P < 0.01, ***P < 0.001 and ****P < 0.0001. (E) Left, mitochondrial membrane potential as assessed by TMRE fluorescence in CD8^+^ T cells treated with 100 ng/ml CXCL10, 25 nM Rapamycin, 10 μM GSK2837808A, or Control for 48 h. Right, quantification of mean fluorescence intensity for TMRE. *P < 0.05, **P < 0.01 and ***P < 0.001 (F) TNF-α proportion in CD8^+^ T cells after intracellular cytokine staining of CD8^+^ T cells activated with PMA/Ionomycin and BFA/Monensin mixtures for 6 h in the presence of 100 ng/ml CXCL10, 25 nM Rapamycin, 10 μM GSK2837808A, or left untreated. *P < 0.05 and **P < 0.01. (G) *Ex vivo* images of resected tumors (left) and growth curves of tumor volume (right) formed by subcutaneous injection of Hepa1-6 cells with or without 100 μg anti-CXCL10 neutralizing antibody (n = 6 per group). (Scale bar: 1 cm). *P < 0.05 and **P < 0.01.

The mTOR pathway provides a critical link between metabolism and function of T cells 39-42, and LDHA is the key player in this metabolic programming 41. Therefore, we speculated that CXCL10 secreted by hepatocytes plays a role in regulating CD8^+^ T cell glycolysis process through mTOR and LDHA signaling. LDHA is a key enzyme in NAD^+^ and NADH transformation. Therefore, we detected the NAD^+^/NADH ratio after inhibiting mTOR with the mTOR inhibitor Rapamycin (Rapa) (Figure 6A). Results demonstrated that in the presence of CXCL10, NAD^+^/NADH ratio decreased in Rapa-treated CD8^+^ T cells (Figure 6B), suggesting that mTOR inhibition directly affected the role of LDHA in glycolysis process.

Rapa and LDHA inhibitor GSK2837808A (GSK) were used to investigate whether the inhibition of mTOR and LDHA affected the glycolysis process regulated by CXCL10 (Figure 6A). Results demonstrated that CD8^+^ T cells treated with mTOR and LDHA inhibitor significantly reduced CXCL10-induced glucose consumption and lactate production (Figure 6C-D). Furthermore, we evaluated the OXPHOS metabolic activities in CXCL10-treated CD8^+^ T cells after using Rapa and GSK. TMRE analysis revealed that in the presence of Rapa and GSK, severely dampened MMP was partially recovered in CXCL10-treated CD8^+^ T cells (Figure 6E). Overall, these data indicated that mTOR and LDHA inhibition are sufficient to decrease CXCL10-induced glycolysis. To clarify whether mTOR and LDHA inhibition are also sufficient to cause immune phenotype changes promoted by CXCL10, we tested the effects of Rapa and GSK on the expression of TNF-α. As shown in Figure 6F, mTOR and LDHA inhibition decreased TNF-α production stimulated by CXCL10.

Furthermore, sh-NC and sh-Smad4 Hepa1-6 cells were inoculated subcutaneously in C57BL/6 mice and CXCL10 was blocked by CXCL10-neutralizing antibody. Control animals were administered with an isotype control antibody. Notably, we found that neutralizing CXCL10 abolished the tumor-suppressive effects of Smad4 knockdown, indicating that CXCL10 inhibition by Smad4 was crucial for its oncogenic activity (Figure 6G). Moreover, anti-CXCL10 antibody significantly decreased the expression of mTOR, LDHA, and TNF-α in CD8^+^ T cells in tumors detected by immunofluorescence (Figure S3G).

### HCC patients with Smad4-high expression exhibit decreased CD8^+^ T cells infiltration and altered glycolysis

**Figure 7:**
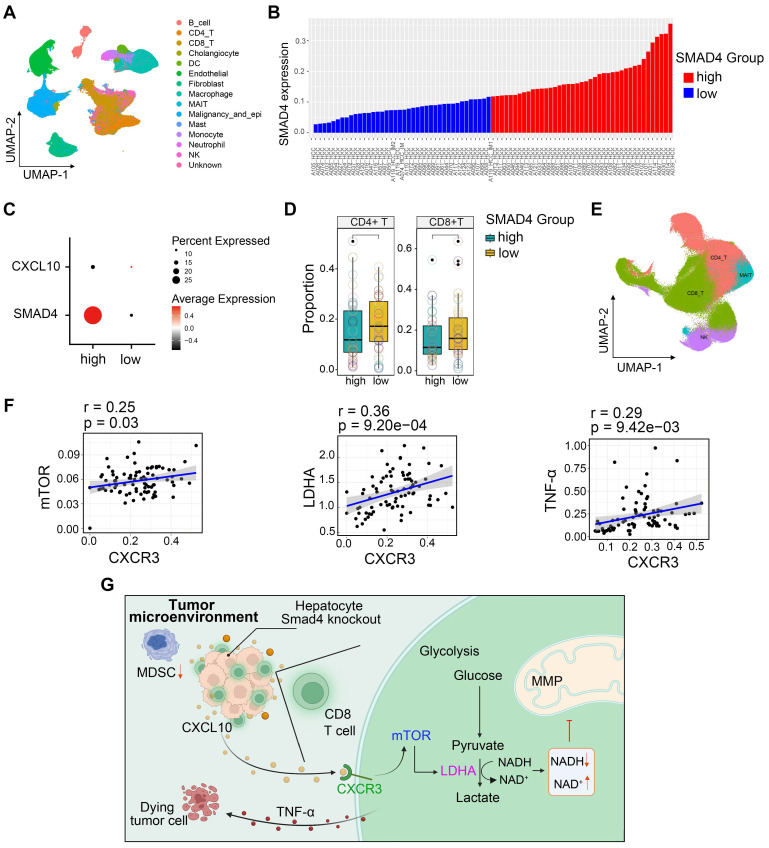
** HCC patients with Smad4-high expression exhibit decreased CD8^+^ T cells infiltration and altered glycolysis.** (A) Uniform manifold approximation and projection (UMAP) plot of broad cell types from all HCC samples (n = 80). (B) The average expression of Smad4 in tumor cells. They were divided into high Smad4 expression group and low Smad4 expression group. (C) Dot plots show the expression of Smad4 and CXCL10 in HCC tumor cells. Dot size indicates the fraction of expressing cells and was coloured according to Z score normalized expression levels. (D) Proportions of CD4^+^ and CD8^+^ T cells in two subgroups (Smad4-high and Smad4-low). (E) UMAP plot of T/NK cell subclusters identified. (F) Correlation between the mRNA levels of CXCR3 and mTOR, LHDA, TNF-α in CD8^+^ T cells. (G) In HCC, Smad4 deletion in hepatocytes leads to increased CXCL10 secretion, thereafter upregulated TNF-α expression and glycolysis in CD8^+^ T cells via the CXCL10/mTOR/LDHA axis.

To extend our findings to human HCC cases, we performed single-cell RNA sequencing (scRNA-seq) analysis of 80 HCC samples to further delineate the functional role of Smad4 in human HCC tumors 28. Single-cell transcriptome profiles of 80 patients with HCC were included. Integrative analysis across this scRNA-seq cohort identified distinct clusters corresponding to canonical markers of indicated cell type (Figure 7A). We used the median Smad4 gene expression level as a cutoff value to split the enrolled patients into Smad4-high and Smad4-low groups (Figure 7B). Results demonstrated that CXCL10 exhibited low expression in tumor cells with Smad4 high expression group, showing a negative correlation between Smad4 and CXCL10 expression (Figure 7C). Additionally, results revealed that patients exhibiting high Smad4 expression within their tumors had a comparatively reduced presence of CD8^+^ and CD4^+^ T cells, suggesting that elevated Smad4 expression promotes a pro-tumoral immune environment (Figure 7D), consistent with conclusions observed in our mouse experiments. Furthermore, we explored the correlation between the CXCL10 receptor CXCR3 and mTOR, LDHA, and TNF-α expression in CD8^+^ T cells. Results demonstrated a significantly positive association between CXCR3 expression and mTOR, LDHA and TNF-α, within CD8^+^ T cells (Figure 7E-F). These findings indicated a diminished anti-tumor immune response in HCC patients with high Smad4 expression and highlighted a positive correlation between the CXCL10/CXCR3 and glycolysis and TNF-α production in CD8^+^ T cells.

## Discussion

Our previous study revealed that hepatocyte-specific Smad4 deletion attenuated CCl_4_-induced liver fibrosis by suppressing hepatocyte proliferation and epithelial-mesenchymal transition (EMT) 15. In the present study, our data further demonstrated that Smad4 deficiency in hepatocytes suppressed hepatocarcinogenesis by increasing of CD8^+^ T cell infiltration. This immunogenic reprogramming was driven by enhanced CXCL10 secretion in hepatocytes, facilitating TNF-α production in CD8^+^ T cells. The specific mechanism involves the promotion of CD8^+^ T cell glycolytic metabolism by CXCL10 through the CXCR3/mTOR/LDHA signaling pathway. A schematic illustration indicated the proposed model of inherent connections between hepatocytes and CD8^+^ T cells in HCC (Figure 7G). These findings demonstrated an important role of Smad4/CXCL10/CXCR3 signaling in fibrosis-related HCC.

Smad4 is a key mediator of the TGF-β pathway and plays complex and contradictory roles during tumorigenesis. Our data revealed that Smad4 was overexpressed in liver tissues of HCC (Figure 1), and that hepatocyte-specific Smad4 deficient mice developed fewer and smaller tumors than control mice. Smad4 knockout could also inhibit the growth of subcutaneous transplanted tumors (Figure 2). Consistent with these findings, Wang *et al.* demonstrated that Smad4 was highly expressed in HBV‑positive HCC patient samples and was associated with poor prognosis 43. Hernanda *et al.* also reported that silencing Smad4 in the human Huh7 cell line decelerated cell proliferation and migration and suppressed implantation tumor growth 22. Furthermore, in the context of HCC bone metastasis, the weakened inhibition of miR-34a on Smad4 promoted the expression of downstream bone metastasis-related genes such as connective tissue growth factor (CTGF) and interleukin-11 (IL-11) 44. However, Smad4 was initially identified as a candidate tumor suppressor gene, whose inactivation may lead to pancreatic cancer (PDAC) 45. Similar situations have also been shown to occur in colorectal and prostate cancers 46, 47. Consequently, the function of Smad4 in regulating tumor progression may be dependent on tumor type, cell type and TEM.

In the TME, Smad4 acts indirectly on anti-tumor immune response by regulating the transcription of multiple chemokines. Our study showed that Smad4 deletion in hepatocytes enhances TNF-α production and glycolysis of CD8^+^ T cells via CXCL10 secretion (Figure 3**)**. Similar to this study, Smad4 silencing in epithelial cells promoted the expression of CCL20, thereby enabling susceptibility to colitis-associated cancer 48. Another study showed that epithelial Smad4 deficiency increased the stemness of gastric cancer cells via CXCL1, which functionally suppressed the function of dendritic cells (DC) and altered the expression of immune checkpoint molecules 4-1BB ligand (4-1BBL) and programmed death-ligand 1 (PD-L1) 49.

CXCL10 derived from tumor provides a key link between tumor cells and CD8^+^ T cells 50, 51 and promotes anti-tumor immune responses 52. Inhibition of lysine-specific demethylase 4C (KDM4C) augments CD8^+^ T cell-mediated antitumor immunity in lung carcinoma by activating CXCL10 transcription 53. Interferon regulatory factor 1 (IRF-1) derived from tumor recruits and activates immune cells to exert an anti-tumor effect on HCC through CXCL10/CXCR3 axis 50. Our study provides direct evidence that Smad4 deletion in hepatocytes regulates CD8^+^ T cell infiltration and activity by increasing CXCL10 secretion (Figure 4). Consistent with this, our further research showed that CXCL10 derived from hepatocytes facilitates glucose metabolism in CD8^+^ T cells, as reflected in higher glycolytic capacity, glucose consumption, lactate production and ATP levels compared with the control group (Figure 5).

Glucose metabolism plays a pivotal role in the regulation of CTL responses. Because effector CD8^+^ T cells undergo extensive proliferation upon antigen stimulation, they require a high glycolytic flux to sustain the bioenergetic demands and provide building blocks for cellular biomass 54. Moreover, glucose metabolism is closely linked to mTOR signaling 39 and T cell cytotoxicity 38. Our results indicated that CXCL10-treated CD8^+^ T cells showed an increase in glycolysis and TNF-α expression, and mTOR and LDHA inhibitors significantly reversed this increasing trend (Figure 6). Although the chemotaxis and differentiation effects of CXCL10 on CD8^+^ T cells have been reported previously 50, 55, 56, this study revealed a previously unknown role of CXCL10 in regulating CD8^+^ T cell effects and metabolic reprogramming. Future studies are required to investigate the *in vivo* immunotherapeutic relevance of the proposed mechanism and determine whether Smad4 inhibition on CXCL10 expands beyond CD8^+^ T cells *in vivo*, affecting other cells within the TME.

In conclusion, this study demonstrated that Smad4 expression in hepatocytes plays a crucial role in HCC. Smad4 deletion in hepatocytes alleviates fibrosis-related hepatocarcinogenesis and increases CD8^+^ T cell infiltration by stimulating CXCL10 secretion, thereby inhibiting HCC progression. Collectively, Smad4 may represent a potential candidate target for the prevention and targeted therapy of HCC, consolidating the preclinical foundation for HCC therapeutic strategies.

## Supplementary Material

Supplementary materials and methods, figures and table.

### Funding

This work was supported by the National Natural Science Foundation of China (81972689), the Natural Science Foundation of Beijing (7232102) and Capital Health Development Research Project (2022-2-4084).

### Author contributions

Jinhua Zhang designed this study. Xin Xin, Xuanxuan Yan and Ting Liu conducted experiments. Xin Xin, Jinhua Zhang and Lingling Hou performed data analysis and wrote the manuscript. Xinlong Yan and Fanxin Zeng performed public datasets analysis. Zhao Li, Zuyin Li and Zhuomiaoyu Chen provided the clinical samples and performed single-cell sequencing data analysis.

### Data availability statement

All data generated or analyzed during this study are included in this article and its online supplementary material. Further inquiries can be directed to the corresponding author.

## Abbreviations

HCC: hepatocellular carcinoma
DEN: diethylnitrosamine
CCl_4_: carbon tetrachloride
OXPHOS: oxidative phosphorylation
ECAR: extracellular acidification rate
OCR: oxygen consumption rate
TCA: tricarboxylic acid
ETC: mitochondrial electron transport chain
