# Supplementary material for: Hepatocyte-specific Smad4 deficiency inhibits hepatocarcinogenesis by promoting CXCL10/CXCR3-dependent CD8+- T cell-mediated anti-tumor immunity
Source: Theranostics. 2024 Sep 9;14(15):5853–68. doi: 10.7150/thno.97276 (PMC11426237; doi:10.7150/thno.97276)
Supplement: Supplementary file 1 — Supplementary materials and methods, figures and table. [file thnov14p5853s1.pdf]

Supplementary materials and methods

Cell culture and treatments

Hepa1-6 cells (ATCC, Manassas, VA, USA) were cultured in Dulbecco's modified Eagle's medium (DMEM) supplemented with 10% fetal bovine serum (FBS) and 1% penicillin/streptomycin. Cells were cultured at 37 °C with 5% CO_2. Hepa1-6 cells were treated with 100 ng/mL IFN-γ (Sino Biological, Beijing, China) for 48 h.

Lentivirus infection

Hepa1-6 cells were transfected by Smad4-targeting lentiviral vector (sh-Smad4) and control Lentivirus (sh-GFP) supplemented with HitransG transfection reagent (Genechem, Shanghai, China). Infected Hepa1-6 cells were cultured with 2 μg/mL puromycin (InvivoGen, San Diego, CA, USA) to obtain the sh-Smad4 stable cell line.

Cell viability assay (MTT)

5×10^3 Hepa1-6 cells with sh-Smad4 and control cells were respectively seeded into 96-well plates in 100 μL medium. The viability was detected by 3-(4,5-dimethylthiazol-2-yl)-2,5-diphenyl-tetrazolium bromide (MTT) method according to the manufacturer's protocols. The OD value of cells was analyzed at 24 h, 48 h and 72 h, respectively.

Wound-healing assays

The Hepa1-6 cells and its sh-Smad4 cells were cultured in 6-well plate and allowed to grow until 90% confluent. The cell layer was scratched with a 200 μL pipette tip. After scratching, cells were washed with serum-free medium. The scratch areas were photographed at 0 h and 72 h, respectively. Quantification of wound healing was performed using Image J software.

Co-culture assays

Hepa1-6 cells were co-cultured with pretreated $CD8^+$ T cells at a 1:3 ratio in 24-well plates, in the absence or presence of anti-CXCL10 neutralizing antibody (20 μg/ml) or control antibody, for 24 h. Subsequently, TNF-α levels in the $CD8^+$ T cells were determined by FACS analysis.

Western blot

Cultured cells and dissected tissues were collected. Total protein was extracted using RIPA lysis buffer (Beyotime, Shanghai, China) containing a protease inhibitor cocktail (Biotool, Houston, TX, USA). Cytoplasmic and nuclear proteins were extracted using a specialized kit (Sangon Biotech, Shanghai, China). Samples were incubated at 99°C for 5 min and separated by electrophoresis on a 10% SDS-PAGE gel at 115 V for 1.2 h. Proteins were transferred to a PVDF membrane at 200 mA for 1 h. Membranes were blocked with 5% milk in TBST for 1 h and incubated overnight at 4 °C with anti-Smad4 (ProteinTech, Chicago, IL, USA) and anti-GAPDH (Affinity Biosciences, Cincinnati, OH, USA) antibodies. HRP-conjugated goat anti-mouse IgG and goat anti-rabbit IgG were used as secondary antibodies. Blots were scanned using a Clinx Science Instrument.

Quantitative real-time polymerase chain reaction (RT-qPCR)

Total RNA was isolated from liver tissues and cells using TRIzol reagent (Invitrogen, Carlsbad, CA, USA) according to the manufacturer's instructions. cDNA was synthesized using a Primescript RT Master Mix Kit (MedChemExpress, Princeton, NJ, USA). qPCR was performed in duplicate with a SYBR Premix Ex TaqTM Kit

(MedChemExpress, Princeton, NJ, USA). Data were analyzed using the $2^{-\Delta\Delta Ct}$ method and normalized to the expression of GAPDH.

ELISA

Hepa1-6 cells with sh-Smad4 and control cells were incubated with IFN-γ (100 ng/mL) for 48 h. After activation, the cells were rinsed with PBS and cultured in fresh serum-free medium. After 48 h, the supernatant was harvested and CXCL10 were quantified using enzyme-linked immunosorbent assay (ELISA) kits (Cloud-Clone Corp, Wuhan, China) according to the manufacturer's protocol.

Glucose, lactate, and ATP measurement

$CD8^+$ T Cells were activated in 96-well plates that were pre-coated with anti-CD3 (2 μg/ml) and soluble anti-CD28 (1 μg/ml) antibody. Activated $CD8^+$ T cells were stimulated with recombinant CXCL10 with or without AMG487 (5 μM), Rapamycin (25 nM), and GSK2837808A (10 μM). Supernatants were collected and analyzed for glucose and lactate using glucose assay kit, lactate assay kit, and ATP assay kit, respectively (Nanjing Jiancheng Bioengineering Institute, Nanjing, China).

NAD^+/NADH ratio

2.5×10^6 $CD8^+$ T cells were cultured in the presence of 100 ng/ml CXCL10 with or without 5 μM AMG487, 25 nM rapamycin and 10 μM GSK2837808A for 48 h. Following the manufacturer's instructions, NAD^+/NADH ratio was determined using a commercially available kit (Beyotime Biotechnology, Shanghai, China).

Quantification of mitochondrial membrane potential

2.5×10^6 $CD8^+$ T cells were cultured in the presence of 100 ng/ml CXCL10 with or without 5 μM AMG487, 25 nM rapamycin and 10 μM GSK2837808A for 48 hours, and then stained with 200 nM TMRE (Beyotime Biotechnology, Shanghai, China) at 37 °C for 30 minutes. cells were washed twice with PBS, and immediately analyzed on the flow cytometer.

Establishment of transplanted tumor model

C57BL/6 mice and nude mice were injected subcutaneously into the abdomen region with 1×10^6 Hepa1-6 cells in 200 μl sterilized phosphate-buffered saline. Tumor size was measured every 3 days by caliper. Tumor volume = ($width^2$ × length) / 2. After 2 weeks, mice were sacrificed by cervical dislocation under anesthesia. Tumor tissues were harvested for single cell suspension preparation or OCT embedding. Frozen slices are prepared. The 100 μg CXCL10 neutralizing antibody (Invitrogen, Carlsbad, CA, USA) or control antibody. was injected i.p. once a week.

Flow cytometry analysis

Single-cell suspensions were collected from liver tissues and spleen tissues respectively, and then incubated with FITC-labelled anti Gr1, APC-labelled anti F4/80, Percp-labelled anti CD11b, APC-labelled anti CD4 and FITC-labelled anti CD8 (BD Biosciences, San Diego, CA, USA) antibody. Cells were collected on a FACSCalibur (BD Biosciences, San Diego, CA, USA) and analyzed by FlowJo software (TreeStar, Ashland, OR USA).

Public database analysis

Transcriptomic data and clinical survival information were obtained from the Gene

Expression Omnibus (GEO) for a cohort of patients with HCC (GEO Series Accession No: GSE 76427, GSE 14520). The dataset includes both gene expression profiles and follow-up survival data.

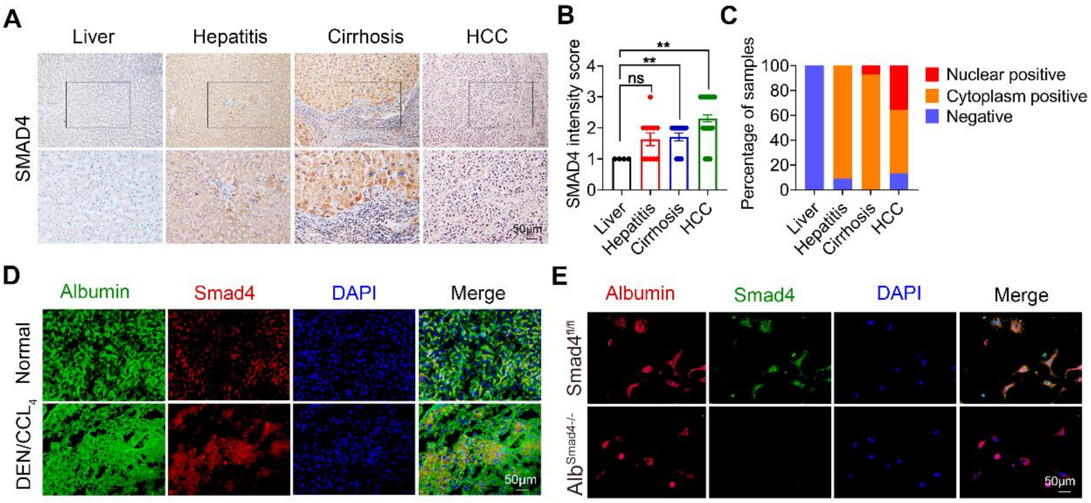
A
Liver
Hepatitis
Cirrhosis
HCC
SMAD4
50μm
B
SMAD4 intensity score
ns
**
**
C
Percentage of samples
Nuclear positive
Cytoplasm positive
Negative
D
Albumin
Smad4
DAPI
Merge
Normal
DEN/CCL4
50μm
E
Albumin
Smad4
DAPI
Merge
Smad4fl/fl
AlbSmad4-/-
50μm

Fig. S1 Smad4 expression is upregulated in human HCC progression stage.

(A) Representative IHC images of Smad4 in human normal liver (n = 5), hepatitis (n = 11), cirrhosis (n = 12), and HCC (n = 45) samples, (Scale bar: 50 μm). (B) Quantification of Smad4 intensity score. **P < 0.01. (C) Smad4 status among different liver specimen types. (D) Representative double staining for albumin (green) and Samd4 (red) in normal liver specimens and DEN/CCl_4-induced liver tissue. (Scale bar: 50 μm). (E) Double staining of albumin (red) and Smad4 (green) in primary hepatocytes (scale bars: 50 μm).

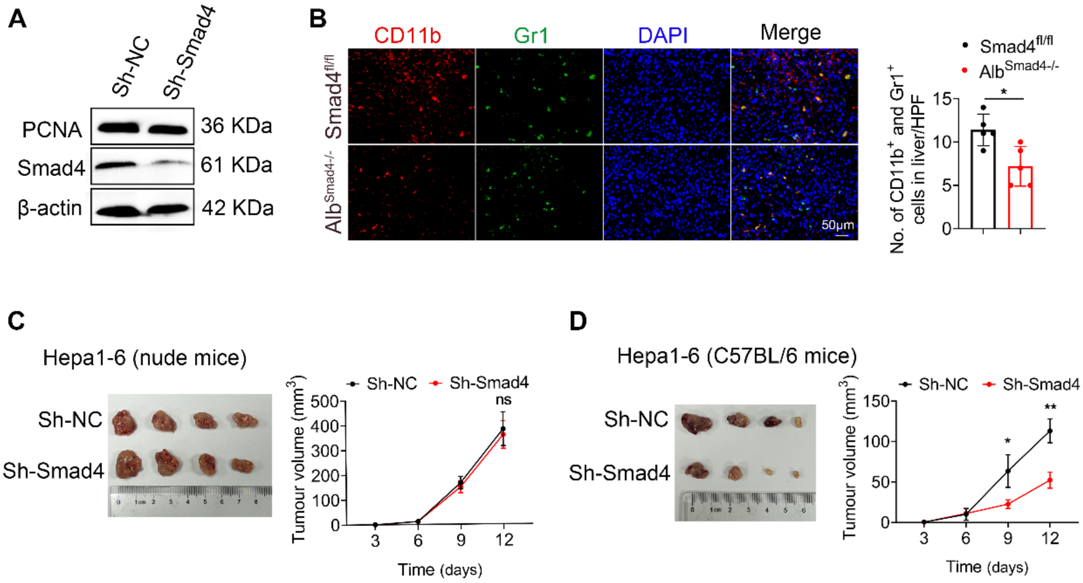
A
Sh-NC
Sh-Smad4
PCNA
36 KDa
Smad4
61 KDa
β-actin
42 KDa
B
CD11b
Gr1
DAPI
Merge
Smad4fl/fl
AlbSmad4-/-
50μm
Smad4fl/fl
AlbSmad4-/-
No. of CD11b+ and Gr1+ cells in liver/HPF
C
Hepa1-6 (nude mice)
Sh-NC
Sh-Smad4
Tumour volume (mm3)
Time (days)
ns
D
Hepa1-6 (C57BL/6 mice)
Sh-NC
Sh-Smad4
Tumour volume (mm3)
Time (days)

Fig. S2. **Hepatocyte specific Smad4 knockout reduced tumor growth in C57/BL6 mice but not in nude mice.**

(A) Western blot analysis of PCNA protein levels in Sh-NC and Sh-Smad4 Hepa1-6 cells. (B) Representative staining and statistical analysis of the co-localization of $CD11b^+$ and $Gr\text{-}1^+$ cells in DEN/CCl_4-induced HCC tissues. (Scale bars: 50 μm). $^{**}P < 0.01$. (C) Ex vivo images of resected tumors (left) and growth curves of tumor volume (right) in nude mice (Scale bars: 1 cm). (n = 4 per group). (D) Ex vivo images of resected tumors (left) and growth curves of tumor volume (right) in C57BL/6 mice (Scale bars: 1 cm). (n = 4 per group). $^{*}P < 0.05$ and $^{**}P < 0.01$.

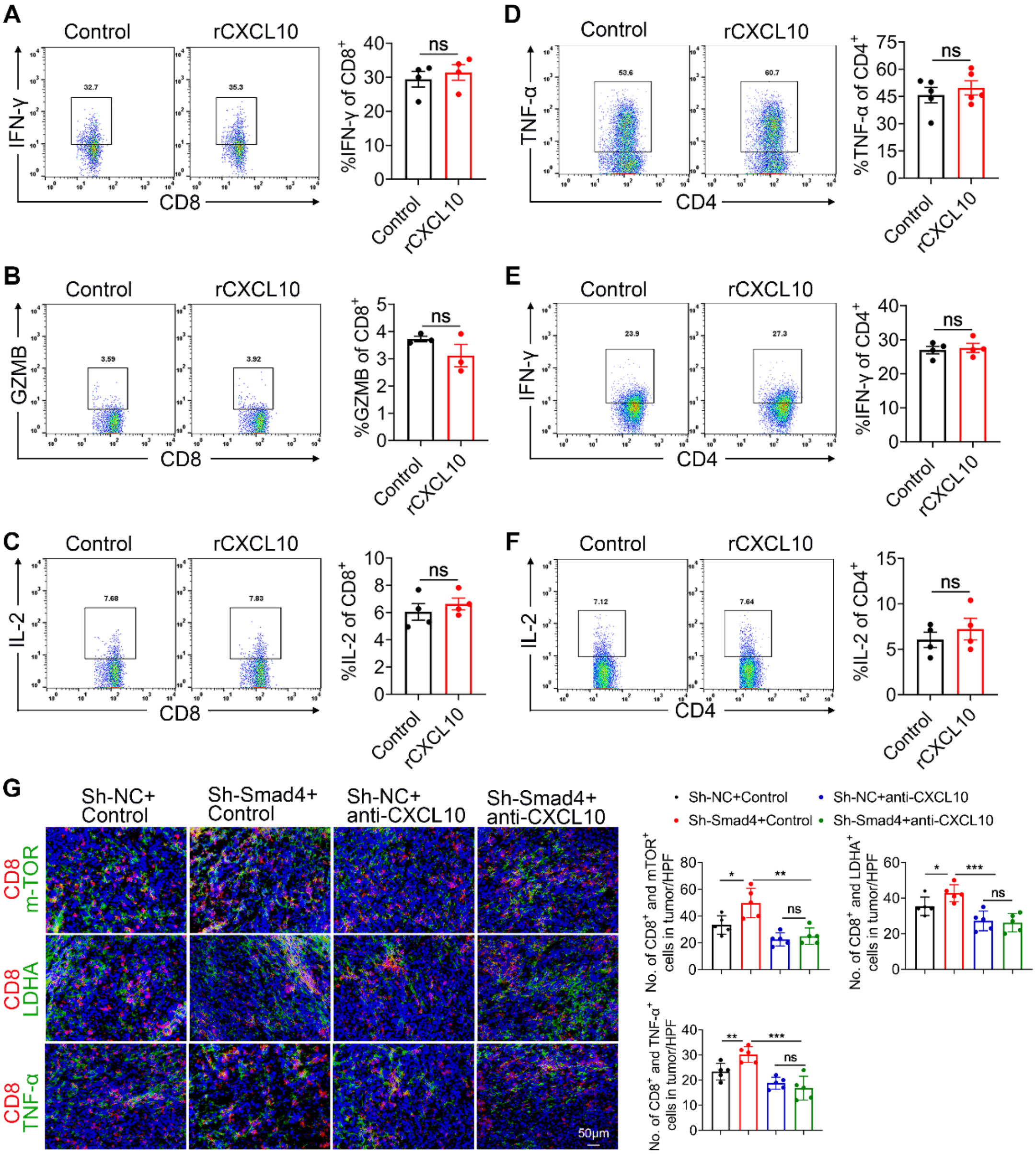
A
Control
rCXCL10
IFN-γ
CD8
%IFN-γ of CD8+
ns
B
GZMB
%GZMB of CD8+
C
IL-2
%IL-2 of CD8+
D
TNF-α
CD4
%TNF-α of CD4+
E
%IFN-γ of CD4+
F
%IL-2 of CD4+
G
Sh-NC+ Control
Sh-Smad4+ Control
Sh-NC+ anti-CXCL10
Sh-Smad4+ anti-CXCL10
CD8 m-TOR
CD8 LDHA
CD8 TNF-α
50μm
Sh-NC+Control
Sh-Smad4+Control
Sh-NC+anti-CXCL10
Sh-Smad4+anti-CXCL10
No. of CD8+ and mTOR+ cells in tumor/HPF
No. of CD8+ and LDHA+ cells in tumor/HPF
No. of CD8+ and TNF-α+ cells in tumor/HPF

Fig. S3 Hepatocyte-derived CXCL10 has no impacts on GZMB, IFN-γ, and IL-2 expression in $CD8^+$ and $CD4^+$ T cell.

(A) IFN-γ, (B) GZMB, and (C) IL-2 levels in $CD8^+$ T cells after intracellular cytokine staining of $CD8^+$ T cells activated with PMA and ionomycin for 6 h in the presence of 100 ng/ml CXCL10 or left untreated. (D) TNF-α, (E) IFN-γ, (F) and IL-2 levels in $CD4^+$ T cells after intracellular cytokine staining of $CD4^+$ T cells activated with PMA/Ionomycin and BFA/Monensin mixtures for 6 h in the presence of 100 ng/ml CXCL10 or left untreated. (G) Representative double staining and statistical analysis for CD8 (red) and mTOR, LDHA and TNF-α (green) in hepa1-6 tumors treated with anti-CXCL10 neutralizing antibody. (Scale bar: 50 μm). *$P < 0.05$, **$P < 0.01$ and ***$P < 0.001$.

Table S1. Real-time PCR primer sequences.

Gene	Species	Primer sequence (5’-3’)
CCL9	Mouse	F: CAGATTGCTGCCTGTCCTAT<br>R: CTGAACTCTCCGATCACTGG
CCL17	Mouse	F: GTACCATGAGGTCACTTCAGA<br>R: CCTTCTTCACATGTTTGTCTTT
CCL20	Mouse	F: CGACTGTTGCCTCTCGTACA<br>R: AGGAGGTTCACAGCCCTTTT
CXCL5	Mouse	F: TCCAGCTCGCCATTCATGC<br>R: TTGCGGCTATGACTGAGGAAG
CXCL9	Mouse	F: CCTAGTGATAAGGAATGCACGATG<br>R: CTAGGCAGGTTTGATCTCCGTTC
CXCL10	Mouse	F: AGTGCTGCCGTCATTTTCTG<br>R: TCAACACGTGGGCAGGATA
GLUT1	Mouse	F: GCAGTTCGGCTATAACACTGG<br>R: GCGGTGGTTCCATGTTTGATTG
HK2	Mouse	F: CTAAGGGGTTCAAGTCCAGTGG<br>R: AGACCAATCTCGCAGTTCTGA
PKM2	Mouse	F: TCGCATGCAGCACCTGATT<br>R: CCTCGAATAGCTGCAAGTGGTA
LDHA	Mouse	F: CAAAGACTACTGTGTAACTGCGA<br>R: TGGACTGTACTTGACAATGTTGG
GAPDH	Mouse	F: TCAATGAAGGGGTCGTTGAT<br>R: CGTCCCGTAGACAAAATGGT
